# Self-sorted Oligophenylvinylene and Perylene Bisimide Hydrogels

**DOI:** 10.1038/s41598-017-08644-0

**Published:** 2017-08-21

**Authors:** Ana M. Castilla, Emily R. Draper, Michael C. Nolan, Christopher Brasnett, Annela Seddon, Laura L. E. Mears, Nathan Cowieson, Dave J. Adams

**Affiliations:** 1 Department of Chemistry, University of Liverpool, Crown Street, Liverpool, L69 7ZD UK; 2 School of Chemistry, WestCHEM, University of Glasgow, Glasgow, G12 8QQ UK; 3 School of Physics, HH Wills Physics Laboratory, Tyndall Avenue, University of Bristol, Bristol, BS8 1TL UK; 4 Bristol Centre for Functional Nanomaterials, HH Wills Physics Laboratory, Tyndall Avenue, University of Bristol, Bristol, BS8 1TL UK; 5 Diamond Light Source Ltd, Harwell Science and Innovation Campus, Didcot, OX11 0QX UK

## Abstract

We describe two component hydrogels with networks composed of self-sorted fibres. The component gelators are based on 1,4-distyrylbenzene (OPV3) and perylene bisimide (PBI) units. Self-sorted gels can be formed by a slow decrease in pH, which leads to sequential assembly. We demonstrate self-sorting by NMR, rheology and small angle X-ray scattering (SAXS). Photoconductive xerogels can be prepared by drying these gels. The wavelength response of the xerogel is different to that of the PBI alone.

## Introduction

Small molecule organic optoelectronic materials provide an interesting counter-point to polymeric or inorganic materials^1–5^. Self-assembly is a widely-used strategy for the organisation of electron-donor and electron-acceptor molecules into larger fibrous structures. The nature of these fibrous structures is key to the charge-transport properties of the materials obtained^6–9^. Low molecular weight gelators (LMWG) have proven useful in this context since they usually self-assemble into one-dimensional fibres, which then bundle up together and entangle forming 3D networks^10^. Thus, combining different gelators with donor and acceptor properties provides a method to prepare optoelectronic materials^7, 8, 11–16^. Mixing two types of gelators can result in co-assembly, where both gelators are present within the same individual fibres, or self-sorting, where only one type of gelator is present in each individual fibre^7, 13, 15, 17–22^, or sometimes somewhere between the two cases. Self-sorted fibres have been hypothesised to provide a better phase separation between donor and acceptor moieties and intimate contacts (at the nodes of the 3D network) between the two phases. These are both requirements for efficient charge generation and transport properties^2, 5^. The morphology of the networks formed, type and number of contacts between the acceptor and donor fibres, are also critical for the photoconductive properties of the film obtained^23^.

A number of two component self-assembled systems, comprising an electron-donor unit and an electron-acceptor unit, have been described for the preparation of self-assembled fibres for optoelectronics^7, 8, 11, 13, 15, 24–26^. In the case of gels, both co-assembled^17^ and self-sorted^15, 18, 27^ systems have been reported, generally in organic solvents, such that organogels are formed. In these cases, a change in temperature is typically used to trigger self-assembly, and self-sorting or co-assembly is driven by differences in solubility or structural match/mismatch of the component gelators.

**Figure 1:**
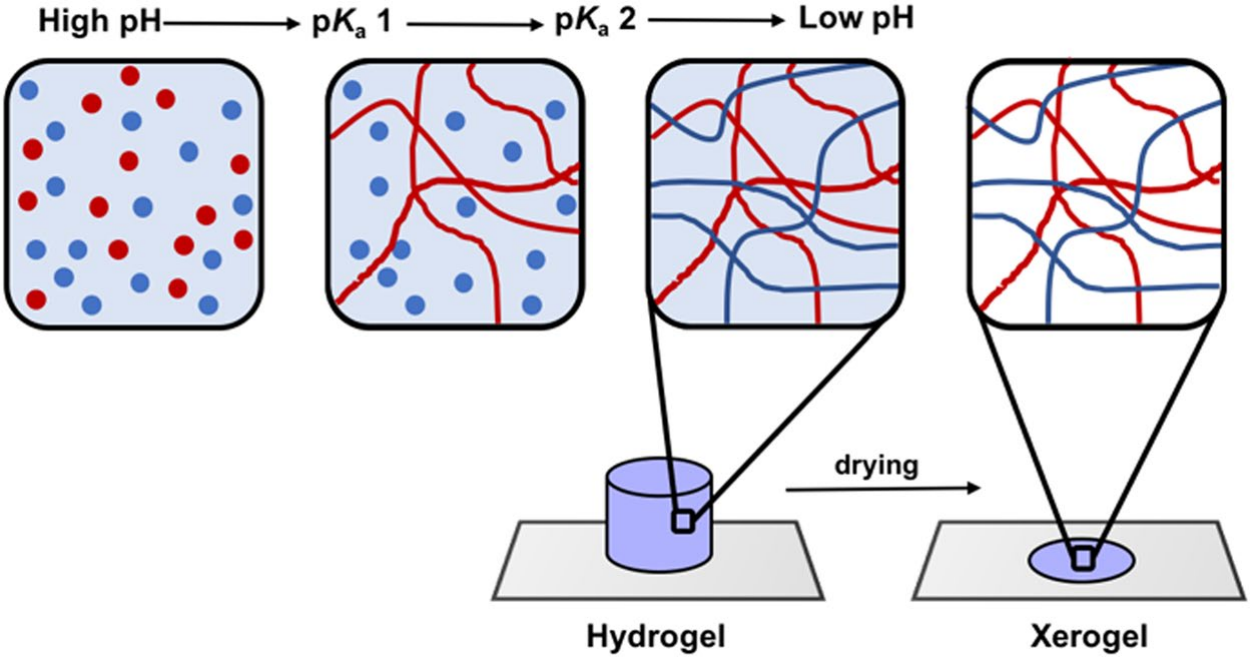
Cartoon showing the sequential self- assembly of two gelators driven by differences in their p*K*_a_ values. Self-sorting of the two gelators results in a hypothetical self-sorted fibrous network. On drying, a self-sorted xerogel will be formed.

As an alternative approach, we are investigating functionalised peptides as hydrogelators, where the trigger for gelation is a change in pH^28^. As such, differences in the p*K*_a_ of the terminal carboxylic acid of the gelators in two component systems, along with a gradual pH change, result in self-sorted systems (Fig. 1)^29, 30^. Recently, we described a mixed system whereby the inclusion of an electron donor, a stilbene-based gelator, into a system containing a perylene bisimide (PBI)-based gelator was shown to affect the absolute wavelength at which a xerogel (a dried gel) became photoconductive^19^. We have also shown that kinetic control over a two component self-assembling mixture can be used to vary the optoelectronic properties of the system^31^.

**Figure 2:**
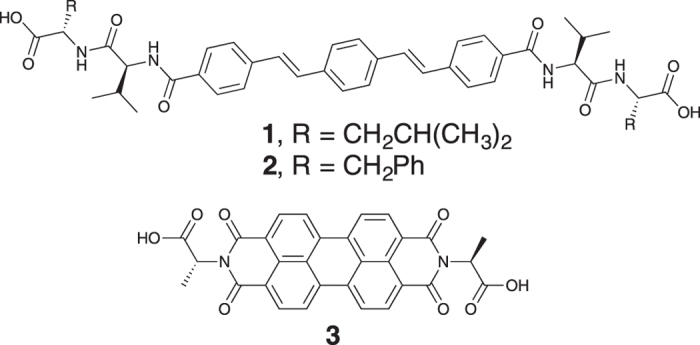
Structures of the two OPV3-based gelators (**1** and **2**) and the PBI-based gelator (**3**) used in this work.

In this work, we set out to investigate the self-sorting behaviour and donor semiconducting abilities of two oligo(*p*-phenylenevinylene)s (OPV3)-based hydrogelators (**1** and **2**)^32^, when mixed with the PBI-based hydrogelator (**3**)^33^ (Fig. 2). OPV3s are well-established electron donor units, whose optoelectronic properties have been shown to be sensitive to intermolecular interactions and molecular organisation. Excitation energy transfer processes have been observed within OPV-based 1D self-assemblies as well as from OPVs to suitable acceptor units^11, 34–37^. In particular, a number of self-assembled systems containing PBI and OPV small molecule components have been reported, where energy transfer has been shown to occur between the components. These include PBI-OPV dyads, arrays of PBIs and OPVs, aggregates, or gels^38–42^. Although a few reports describe co-assembled OPV and PBI mixtures^17, 43, 44^, to the best of our knowledge, self-sorted aggregates in water have not been reported. A system of self-sorted fibres (Fig. 1) however, could provide an interpenetrating network of donors and acceptors, which has been suggested to be required for effective ambipolar charge transport^44^.

## Results and Discussion

To create two component networks with photoinduced energy transfer, we have investigated the mixtures of OPV3-based gelators, **1** or **2**, with PBI-based gelator (**3**) (Fig. 2). We recently described the gelation of these three gelators as single components; in all cases, hydrogels can be formed by preparing an aqueous solution of any of these gelators at high pH and then lowering the pH^32, 33^. We use the hydrolysis of glucono-δ-lactone (GdL) to gluconic acid as the means of controllably lowering the pH^45, 46^. We showed that both **1** and **2** form hydrogels that undergo syneresis over time, expelling some of the water trapped in the network^32^. The gels formed from **1** were much more susceptible to this behaviour than those formed from **2**. We have previously shown that we can prepare photoconductive films by either drying a solution of PBI-gelator **3** at high pH, or by drying the gel formed at low pH^19, 33, 47^.

Solutions of either **1** + **3** or **2** + **3** were prepared by mixing solutions at high pH of the individual gelators such that the final concentration was 5 mg/mL of each gelator. For convenience, the relative concentrations were not varied. All the gelators (**1**, **2** and **3**) form gels at this concentration as single components. At high pH, the mixture of **2** + **3** was significantly more viscous than that of **1** + **3** (Fig. S1, Supporting Information) implying that worm-like micelles are present^48^; this correlates with the presence of worm-like micelles in the solutions of **2** as we have previously observed^32^. Tow-component gels were formed upon addition of an aliquot of each solution to GdL.

The slow, reproducible hydrolysis of GdL allows for the gelation process to be followed by a number of techniques^19, 45^. The self-assembly of the gelators is driven by protonation of the terminal carboxylate groups upon acidification of the solution. Each component starts to self-assemble into fibres when the apparent p*K*_a_ of the corresponding carboxylic acid is reached^19, 28^. We have previously found two distinct apparent p*K*_a_ values for both the OPV3-based gelator **1** (6.8, 6.2) and the PBI-based gelator **3** (6.6, 5.4) and only one for the OPV3-based gelator **2** (7.2)^19, 32^. We would therefore expect four different p*K*_a_ values for the two component mixture **1** + **2** and three for **2** + **3**. However, by titration of high pH solutions of **1** + **3** with HCl, only three apparent p*K*_a_ values were identified (as shown by the plateaus in the pH in Fig. S2, Supporting Information). We attribute this result to the proximity of the expected p*K*_a_ values of the individual gelators in the mixture, not allowing for a clear differentiation between them during titration. Titration of the high pH solution of **2** + **3** did result in the identification of the three expected p*K*_a_ values (Fig. S2, Supporting Information).

**Figure 3:**
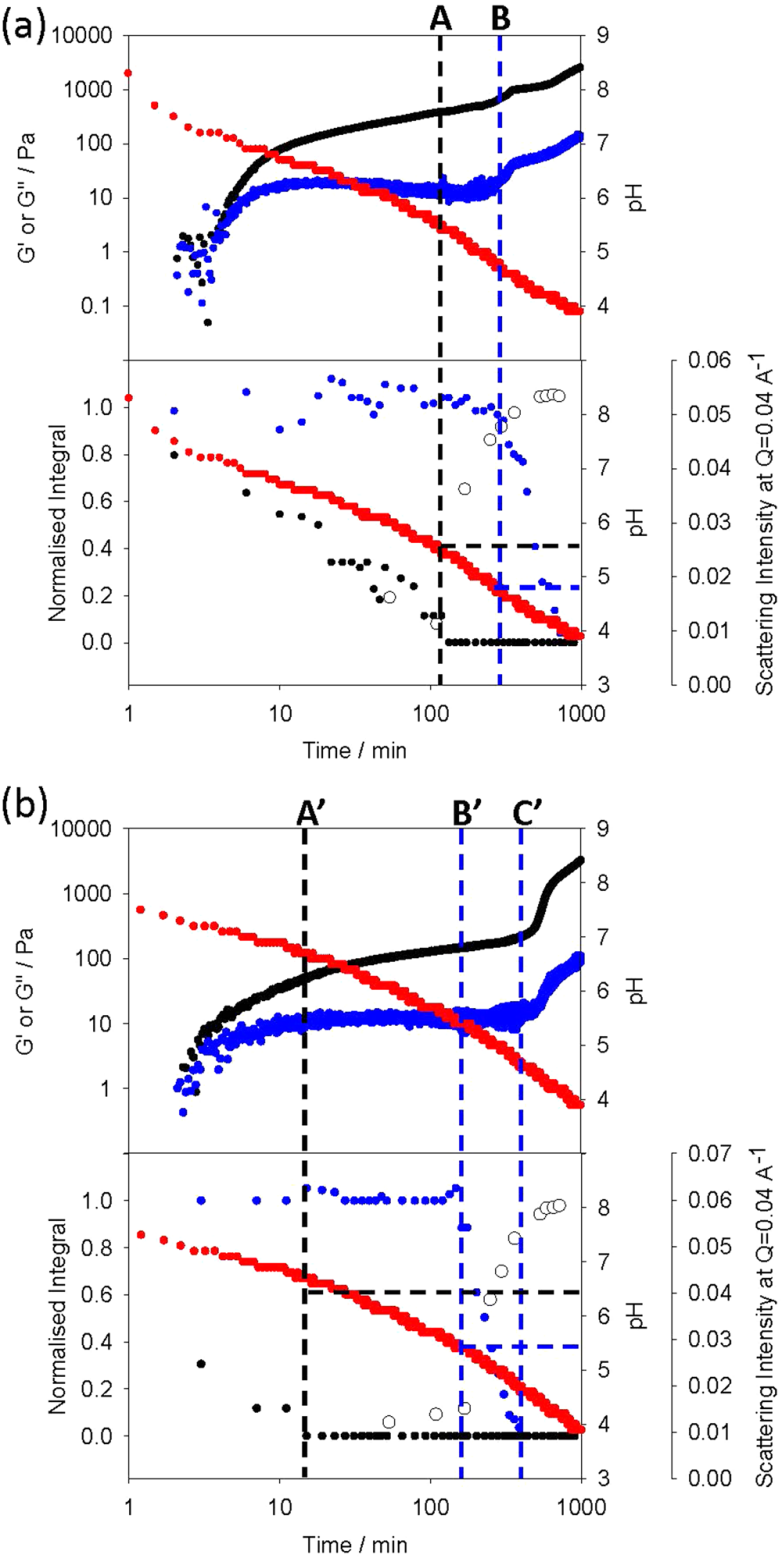
Plots showing the evolution of the gel networks of the two component systems with time, monitored with different techniques: (**a**) **1** + **3**, (**b**) **2** + **3**. For (**a**) and (**b**), the top plots show the evolution of pH (red data) and rheological moduli (G′, black; G″, blue). The bottom plots show the evolution of pH (red data), integral of ^1^H NMR signals due to CH groups in **1** (black in (**a**)), **2** (black in (**b**)) and **3** (blue in (**a)** and (**b**)), and SAXS (open circles). Note that the scatter of the NMR data is attributed to the inherent inaccuracy of integrating broad NMR signals (see Fig. S3 Supporting Information). The normalised value was determined from the integration of the components against the internal standard at pD 11 before the addition of GdL.

For both mixtures, the gelation process was monitored by pH measurements, ^1^H NMR spectroscopy, rheology, and small angle X-ray scattering (SAXS) (Fig. 3). Because of the reproducibility of the GdL-triggering method, experiments can be directly compared provided temperature, starting concentration and pH are accurately controlled^19, 28^. The amount of GdL used for each mixture was optimised to ensure that the final pH of the systems was as close as possible. Since the p*K*_a_ values of the gelators are different, this necessitated a screen of varying amounts of GdL. We found that both mixtures undergo sequential self-assembly where the OPV3-gelators (**1** or **2**) associate prior to the PBI-gelator (**3**). Note, the hydrolysis of GdL is base catalysed and hence the initial drop in pH is very quick. As such, because of the time taken to load the samples in the various experiments, the pH data recording starts at 1 minute and hence the data in Fig. 3 start at a pH of around 9. ^1^H NMR spectroscopy provides information on this process since the gelators gradually become NMR-invisible upon gelation^49^. Thus, as the pH of the pre-gelation solution slowly drops over time, the self-assembly of each gelator can be tracked by monitoring the decrease in the integral values of characteristic ^1^H NMR peaks^50^. The plots in Fig. 3 (see also Fig. S3, Supporting Information) show that for both two-component systems, the NMR peak monitored for the OPV3-based gelator (**1** or **2**) disappears prior to that for PBI-gelator (**3**). On addition of GdL to a solution of **1** + **3** (5 mg/mL of each gelator, pH > 10) the integral of the ^1^H NMR signal monitored for **1** (Fig. S3, ESI) immediately starts to decrease, until it has completely disappeared after 132 min (point A in Fig. 3a). At point A, the pH of the mixture is 5.4, which is lower than the pK_a_ of **1** as expected. In contrast, the integral of the ^1^H NMR signal monitored for **3** (Fig. S3, ESI) remains constant and only starts to decrease after 170 min (point B in Fig. 3b) when the pH is 5.2 (around the first p*K*_a_ of **3**). The mixture of **2** + **3** behaves similarly, with a larger time gap between the complete disappearance of **2** (point A’ in Fig. 3b) and the point at which **3** starts to self-assemble (point B’ in Fig. 3b). These results demonstrate sequential self-assembly which implies that self-sorting is occurring during gelation of the two-component systems^28, 51^.

Rheological data further supports the formation of self-sorted gel networks for the mixtures of **1** + **3** and **2** + **3** (we have reported changes in rheology with time many times previously^28, 30, 45^; the low strain and frequency used during this experiment does not appear to significantly affect the progress of gelation and are in the linear viscoelastic (LVE) region of the final gel). In both cases, a gel is formed as the OPV3-gelator self-assembles; the storage modulus (G′) quickly starts to dominate over the loss modulus (G″), with both reaching a plateau once either **1** or **2** has fully self-assembled (as indicated by the complete disappearance of the peaks for these gelators from the NMR spectra, points A and A’ in Fig. 3). After that point, the pH drops further such that **3** starts to self-assemble (points B and B’ in Fig. 3).

We observed a slight difference in behaviour between the two systems described here. When **3** assembles in the mixture of **1** + **3**, there is a slight increase in the rheological moduli, whereas for **2** + **3**, there is no increase at this point (B and B’ in Fig. 3). Instead, the moduli increase at a slightly later time. As for the gels of **1** and **2** alone^32^, the two-components gels (**1** + **3** and **2** + **3**) were observed to undergo syneresis, although over much longer timescales than for the single component systems (≫24 hours). It is possible however, that the increase of the moduli at longer times for the mixture of **2** + **3** is due to the beginning of the contraction of the gel network formed by **2** that later leads to bulk syneresis. For both two component mixtures, the final gel behaves like a typical low molecular weight hydrogel, with the moduli being essentially independent of frequency. Both G′ and G″ were also independent of the strain until >10% (Fig. S4, Supporting Information).

**Figure 4:**
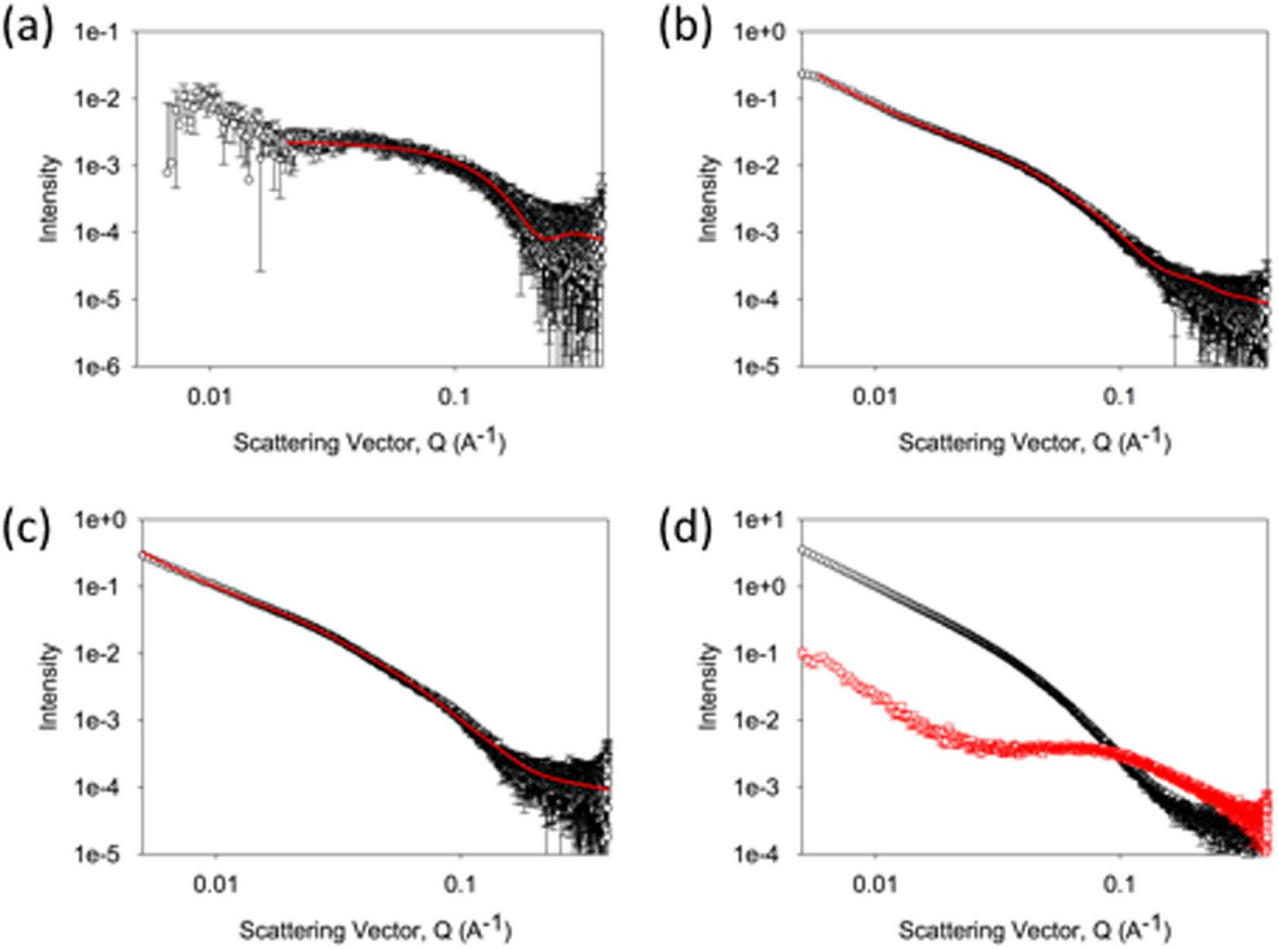
SAXS data and fitting. The scattering is shown for (**a**) a solution of **1**; (**b**) a gel of **1**; (**c**) a gel of **3**; In all cases, the data are shown in black and the fits are shown in red. (d) shows an overlay of the scattering from a solution of **1** + **3** immediately after adding GdL (red) and after 800 minutes (black). Further SAXS data is available in the Supporting Information.

The formation of the gel networks of **1** + **3** and **2** + **3** was further monitored using SAXS. As a reference, we first probed the single-component high pH solutions and low pH hydrogels. The analysis of the SAXS data collected for the single-component systems of **1** and **2** is consistent with the small angle neutron scattering (SANS) studies reported in previous work^32^. The data for the high pH solutions of **1** or **2** alone can be fitted to a combination of flexible cylinder and power law models. For **1**, the high Q region can be fitted to a sphere with a radius of 1.88 ± 0.11 nm, with the remainder of scattering being effectively a power law (Fig. 4a). This can be assigned to a co-existence of free molecules and a very small fraction of worm-like micelles. For **2** alone, at high pH (Fig. S5b, Supporting Information) the radius of the cylinder was fitted as 1.5 ± 0.1 nm, the Kuhn length (which is proportional to the persistence length) fitted to 19.4 ± 4.7 nm, and the length to 121.8 ± 5.8 nm. This indicates that there are worm-like micelles present at high pH^32^.

The scattering data from the gel of **1** alone can be fitted to a combination of flexible cylinder and power law models (Fig. 4b)^32^. The radius of the cylinder was fitted as 2.5 ± 0.2 nm, the Kuhn length (proportional to the persistence length) fitted to 6.2 ± 0.3 nm, and the length to 25.1 ± 0.1 nm. The data for the gel of **2** alone is best fitted by a combination of a power law and a flexible elliptical cylinder model (Fig. S5e, Supporting Information), with a radius of 1.5 ± 0.6 nm, a Kuhn length of 10.7 ± 0.5 nm, a length of 42.7 ± 1.6 nm, and an axis ratio of 2.57 ± 0.2.

The scattering from **3** alone at high pH is very weak, and hence was not fitted (Fig. S5c, Supporting Information; we have previously reported that at lower pH, scattering from worm-like micelles is observed^47^). On gelation, the scattering for **3** alone could be fitted to a flexible cylinder model, which required a degree of polydispersity in radius to optimise the fit (Fig. 4c). The radius of the cylinder was fitted as 4.2 ± 0.1 nm, the Kuhn length (which is proportional to the persistence length) fitted to 30.0 ± 2.3 nm, and the length to 145.0 ± 13.1 nm. This low scattering correlates with the relative sharpness of the peaks in the NMR for **3** as compared to those of **1** and **2** which shows that at pH 11, **3** is less persistently aggregated than either OPV. The data here agree with our previous SANS data^31, 32, 52^, and the work of Martin *et al*.^53^; where worm-like micelles are formed at high pH, the scattering intensity is relatively high. If spherical micelles, or less persistent structures are formed, the scattering at high pH is low.

For the two component systems, we monitored the changes in scattering accompanying the formation of the gel network. Immediately after the addition of GdL to the high pH solutions, the scattering profile of both mixtures **1** + **3** (Fig. 4d) and **2** + **3** (Fig. S5h, Supporting Information) most strongly resembles that of the corresponding OPV3-based gelator alone. This is expected considering that **1** and **2** scatter much more strongly than **3** at high pH (see above). However, by >800 minutes the scattering becomes most similar to that from **3** alone, although with subtle changes associated with the presence of structures arising from either **1** or **2** in the corresponding mixture. This is in agreement with the more intense scattering of the structures in the gel of **3** than those formed from **1** or **2**.

The temporal evolution of structure was studied in both mixtures (**1** + **3** and **2** + **3**) by following the scattering at a Q of 0.04 Å^−1^ (Fig. 3a and b). There was again a difference between systems. For **1** + **3**, the scattering intensity started to increase at point A. This is before **3** has started to disappear from the NMR spectrum. In line with our previous data^19^, we assign this to the formation of worm-like micelles^33, 47^ prior to fibre formation. The intensity continues to change subtly after point B where **3** is assembling into fibres. For **2** + **3**, the intensity of the SAXS increases at B’, where **3** also begins to disappear from the NMR spectrum.

Together, the results of NMR, rheology and SAXS studies demonstrate that the gelators in **1** + **3** and **2** + **3** solutions self-assemble sequentially after the addition of the GdL trigger. In both cases, the OPV3-based gelators (**1** or **2**), which have higher p*K*_a_ values than the PBI-based gelator **3**, start to self-assemble first. This implies that self-sorted fibre networks form the final gel (Fig. 1)^19, 28, 29^. In further agreement, the UV-Vis absorption spectra of the gels of **1** + **3** and **2** + **3** are simply the sum of the spectra of the two components (Fig. S6, Supporting Information). We suggest that the lack of any new peaks in the spectra of the mixed gels is due to the absence of any ground state charge transfer complexes, which would be expected to arise from co-assembled structures. However, we note that because of the highly absorbing nature of the components and the fact that dilution cannot be used as the structures formed are concentration dependent, it is difficult to be certain that the spectra are truly linear sums of the components^38^. We note that co-assembled OPV and PBI mixtures have been shown to have a UV-Vis absorption spectra that differed from a simple sum of that of the components^43^.

**Figure 5:**
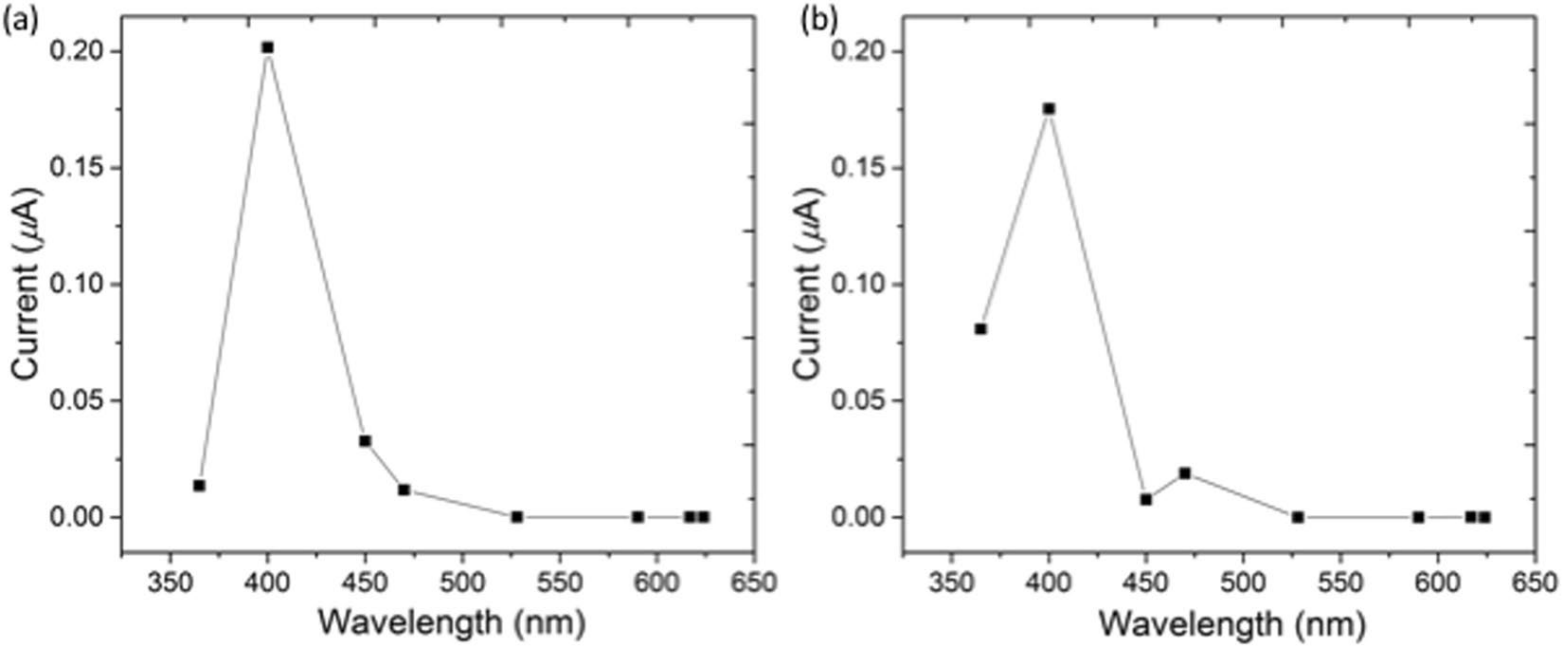
(**a**) Photocurrent at 4 V of the xerogel of **1** + **3** at different wavelengths; (**b**) Photocurrent at 4 V of the xerogel of **2** + **3** at different wavelengths.

Next, we probed the photoconductive response of the xerogels of **1** + **3** and **2** + **3** prepared by drying the pre-formed self-sorted hydrogel networks. The gels were dried in air in a mask such that thin films of defined dimensions were formed^19^. The photoconductivity under illumination was measured by placing silver electrodes on both sides of the film and attaching them to a potentiostat by copper wires (Fig. 1). Linear sweeps between −4 V and 4 V were carried out to enable measurement of the conductivity under illumination, via the change in resistance (Fig. 5). Since syneresis was not observed for the two-component gels over the timescales for gelation and xerogel formation, we do not expect this process to affect the network and hence photoconductivity. We have previously shown that xerogels of **3** alone are photoconductive^33^, with a maximum conductivity when irradiated with wavelengths of less than 400 nm. We were unable to prepare good quality xerogel films of **1** or **2** alone because of their propensity to syneresis^32^.

Both xerogels (**1** + **3** and **2** + **3**) showed the greatest response (i.e. the largest decrease in resistance) when the films were irradiated with a 400 nm LED. Under 365 nm LED illumination, xerogel **2** + **3** showed a significant response whilst xerogel **1** + **3** did not. Above 400 nm, very weak or no photoresponse was observed in either of the xerogels (Fig. 5). Thus, using a self-sorted OPV3-PBI donor-acceptor system results in a shift of the maximum photoresponse from 365 nm in the xerogels of **3** alone^33^ to 400 nm, closer to the visible region. This is in agreement with our previous work on a mixed gel of **3** and a stilbene-based gelator, where the maximum photoresponse was also shifted to 400 nm^19^. Whilst forming the gels for photoconductivity measurements, no shear stress was used. Hence, we expect a random fibrous network, with limited alignment. We note that we have previously shown that it is possible to align the fibres of **3** alone^54^. On doing this, the absolute magnitude of the conductivity changes, but the wavelength response does not. We thus attribute the changes in the wavelength response between systems to the presence of the OPV as opposed to any changes in the degree of alignment.

The photoconductivity in these systems is attributed to the formation of the radical anion (PBI^•−^) of **3** under irradiation. For **3** alone, the xerogel must be irradiated at wavelengths of 400 nm or less to generate the radical anion^33^. For the xerogels of the mixed systems (**1** + **3** and **2** + **3**) the radical anion was detected after irradiation at 365 nm as indicated by the appearance of the characteristic peaks in the UV-Vis spectra at 730, 820 and 1000 nm (Fig. S7, Supporting Information)^33, 55^. At this wavelength, isomerisation of the OPV was also observed, as shown by the decrease in the intensity of the peak at 365 nm. Irradiation at 400 nm leads to formation of the PBI radical anion in both single-component (**3** alone) and two-component (**1** + **3** and **2** + **3**) systems, with no observed isomerisation in the latter (Fig. S8, Supporting Information). In previous work, we suggested the amino acid acts as the donor in systems of **3** alone^47^, whereas for mixed systems the stilbene component could act as electron donors^33^. We presume that the OPVs are similarly able to act as donors. Comparing the systems, it appears that the measured photoconductivities for the mixed systems composed of PBI-gelator (**3**) and OPV3-based gelators are an order of magnitude higher that the conductivity measured for our previous mixed systems composed of **3** and a stilbene(OPV2)-based gelator (data shown in Fig. S10). This could imply that the OPV3s are more efficient donors than the stilbene, although we cannot rule out differences in absolute fibre alignment between the electrodes leading to this apparent increase^54^ or differences in absolute amounts of xerogel used.

## Conclusions

In conclusion, we have shown that we are able to apply our self-sorting methods to a mixture of an OPV3 gelator and a PBI-based gelator. Slightly different assembly processes happen for the two different OPV3 investigated. Along with other recent work^52^, this shows the complexity of this approach. The self-sorting can be followed by a number of techniques, including SAXS and NMR. We highlight again the effectiveness of the GdL hydrolysis approach for allowing comparative data to be collected. To the best of our knowledge, this is the first example where SAXS has been combined with other analytical approaches to prove self-sorting.

Both mixtures of OPV3 and PBI can be used to form photoconductive films. The wavelength range over which the xerogel films are photoconductive is shifted as compared to the PBI alone, showing that the OPV3s are capable of acting as electron-donors.

The demonstration that self-sorting is occurring shows that we are able to control the assembly of the molecules into fibres. Whilst these data are encouraging and show further the potential of this approach, we are unable to prove currently how the self-sorted fibres are interacting with one another. An interpenetrating network is likely to be most effective^44^, but proving this is extremely difficult^29^. To fully understand and optimise these systems, it is necessary to characterise the assembly of fibres. We will address this issue in future work.

## Methods

### Materials

The gelators were synthesised as described previously^32, 33^. De-ionised water was used throughout. All other chemicals were purchased from Sigma Aldrich and used as received.

### Preparation of solutions at high pH

Solutions of **1** or **2**: to a vial containing a pre-weighed amount of the gelator was added NaOH (2 molar equivalents of an aqueous solution) and H_2_O to give a final concentration of the gelator of 5 mg/mL or 10 mg/mL. This solution was vigorously stirred until complete dissolution of the gelator.

Solutions of **3**: to a vial containing a pre-weighed amount of the gelator was added NaOH (1 molar equivalent of an aqueous solution) and H_2_O to give a final gelator concentration of 5 mg/mL or 10 mg/mL. This solution was vigorously stirred until complete dissolution of the gelator.

Solutions of the mixed systems **1** + **3** and **2** + **3** were prepared by mixing a known volume of a solution of either **1** or **2** (10 mg/mL) and the same volume of a solution of **3** (10 mg/mL), so that the resulting solution is 5 mg/mL of each gelator (10 mg/mL overall concentration of gelator). This would usually result in a solution with a pH ≈ 6.5. Since this value is close to the p*K*_a_ values of the components, we increased the pH to remove complications from slight variations in pH at values close to the p*K*_a_s. The pH was therefore increased to 10–11 with a 1 M NaOH aqueous solution.

### Preparation of hydrogels

Gels were prepared by adding glucono-δ-lactone (GdL) to the solutions of the gelators. The mixture was manually swirled and left to stand at room temperature. The amount of GdL added was based on our previous optimisation for the individual gelators^19, 32, 33^:

- 3 mg/mL GdL to 5 mg/mL solution of **1** and 6 mg/mL of GdL to 10 mg/mL solutions of **1**
- 5 mg/mL GdL to 5 mg/mL solution of **2** and 10 mg/mL of GdL to 10 mg/mL solutions of **2**
- 5 mg/mL of GdL to 5 mg/mL solution of **3** and 10 mg/mL of GdL to 10 mg/mL solutions of **3**
- 8 mg/mL of GdL to solution of **1 + 3**
- 10 mg/mL of GdL to solution of **2 + 3**

### pH Measurements

A calibrated FC200 pH probe (HANNA instruments) with a 6 mm × 10 mm conical tip was used for pH measurements (stated accuracy of the pH measurements is ±0.1).

The apparent p*K*_a_ of the gelators in mixtures of **1** + **3** or **2** + **3** were determined *via* titration of the high pH solutions (2 mL of a 10 mg/mL) with a 0.1 M HCl solution. To prevent the gel from forming, the solutions were stirred continuously. pH measurements were recorded after each addition of HCl (typically 3–5 μL) when a stable value was reached (3–10 minutes after addition).

To monitor the pH changes during the gelation process, 2 mL of stock solution of gelator was added to a pre-weighed amount of GdL. After swirling to ensure dissolution of the GdL, the sample was placed in a circulating water bath at 25 °C and the pH measured every 0.5 minutes. The sample was not stirred.

### Dynamic rheological and viscosity measurements

 Rheological and viscosity measurements were performed using an Anton Paar MCR101 or MCR301 rheometer. All data were collected at 25 °C. A parallel plate geometry was used to perform time sweeps, a cone and plate system to perform viscosity measurements and a cup and vane geometry to perform frequency and strain sweeps.

For the time sweeps, 1 mL of a stock solution was added to a plastic vial containing the required amount of GdL. The mixture was homogenised by swirling manually. An aliquot of the solution (450 *μ*L) was then transferred to the bottom plate in the rheometer, the top plate lowered, and the evolution of storage and loss moduli were monitored over time. Once the lower plate was lowered, mineral oil was placed around the system to prevent the solution from drying. For viscosity measurements, stock solutions were prepared at high pH as described above, and 2.1 mL aliquots were transferred onto the plate for the measurement. For frequency and strain sweeps, gels were prepared as described above in plastic (7 mL Sterilin) vials and rheological measurements carried out after 18 hours.

**Time sweeps** were performed on the MCR101 rheometer using a 25 mm plate with a plate gap of 0.8 mm. Tests were performed at an angular frequency of 10 rad s^−1^ and with a strain of 0.5%.

**Frequency sweeps** were performed on the MCR301 rheometer. Frequency scans were performed from 1 rad/s to 100 rad/s under a strain of 0.5%. The shear modulus (storage modulus (G′) and loss modulus (G″)) were read at 10 rad/s. These measurements were done within the viscoelastic region where G′ and G″ are independent of strain amplitude.

**Strain sweeps** were performed on the MCR301 rheometer. Strain sweeps were performed from 0.1% to 100% at a frequency of 10 rad/s. The critical strain was quoted as the point that G′ starts to deviate for linearity and ultimately crosses over the G″, resulting in gel breakdown. Again, this method made sure that 0.5% strain was in the viscoelastic region required for measuring the frequency sweep.

**Viscosity measurements** were performed on the MCR101 rheometer using a cone and plate geometry (CP75 with diameter 75mm and angle 1°). The viscosity of each solution was recorded under the rotation shear rate from 1 to 100 s^−1^.

### NMR spectroscopy

^1^H NMR spectra were recorded at 298 K using a Bruker Avance VIII HD 500 MHz spectrometer. Spectra were processed using Bruker Topspin 3.2.

Stock solutions of **1** + **3** and **2** + **3** were prepared as described above in D_2_O (as opposed to H_2_O) and used to monitor the gelation process by ^1^H NMR. The pH of the resulting solution was adjusted to 10.3 with a NaOD solution (1.0 M) in D_2_O. MeOH (2 *μ*L) or EtOH (2 *μ*L) was then added as an internal standard for the mixture of **1** + **3** or **2** + **3** respectively. 500 *μ*L of the stock solution were added to a NMR tube and the ^1^H NMR recorded to ensure an accurate t = 0 spectrum was recorded and to allow integration of the internal standard against both components. A different NMR tube was loaded with GdL and 500 *μ*L of the stock solution, the mixture manually mixed, and placed as quickly as possible into the NMR spectrometer. The results were plotted using the integrals of the resonance of **1** at 2.12 ppm, the resonance of **2** at 2.75 ppm, and the resonance of **3** at 5.3 ppm. Aliquots of the same stock solutions were used for pH and rheological measurements.

### UV-Vis Spectroscopy and Xerogel Irradiation

Xerogels were prepared on a glass slide and UV-Vis absorption spectra were obtained between 1100–300 nm using an Agilent Cary 60 Spectrophotometer and between 1500–1000 nm using a Shimadzu UV-3600 Spectrophotometer. Samples were irradiated for 10 minutes using single wavelength LEDs of 365 nm (Fig. S7) and 400 nm (Fig. S8) and spectra were collected before and after irradiation. The radical anions persist for hours in the films as we have shown previously^33^. Typically, the irradiation was completed approximately 5 seconds before the UV-Vis measurements were collected. Spectra of samples containing **3** were normalised to the 0–0 vibrational transition of the S_0_-S_1_ absorption (*ca*. 578 nm).

UV-Vis of the gels were measured in 0.1 mm path length quartz demountable cuvettes. 100 *μ*L of pre-gelation solution containing GdL was transferred to the cuvette while still liquid. The cuvette was sealed with Parafilm and the sample allowed to gel overnight before the spectrum was recorded. UV-Vis spectra of the xerogels were collected on glass slides. The gel was formed in a vial as described above, then transferred onto a glass slide and allowed to dry in air overnight to form a thin film xerogel.

### Small Angle X-Ray Scattering

Solutions were prepared as described above and were loaded into 1.56 mm diameter Kapton capillaries. To form the gels, solutions were added to the GdL in a vial and immediately transferred to Kapton capillaries. The capillaries were sealed and held in the X-ray beam on the B21 beamline (Diamond Light Source, Oxfordshire, UK). The beamline operates at a fixed energy of 12.4 keV and camera length of 4.014 m, resulting in a *Q* [*Q* = 4πsin(*θ/2*)/*λ*] range of 0.004 to 0.4 Å^−1^. Measurement times were 10 seconds for the solution samples and 3 seconds for the gels. Background subtraction was carried out using the ScÅtter software (from http://www.bioisis.net/) with a background of the same NaOH concentration as the samples in a Kapton capillary was subtracted from the data.

The data were fitted to customised models in the SasView software package^56^. The models used were either a spherical model, or a model which combined an absolute power law with either a flexible elliptical cylinder or a (Kratky-Porod) flexible cylinder. The *Q*-dependent power law (*Q* ^*−N*^) accounts for the mass fractal contribution to the scattering intensity, which is superimposed on that from the flexible elliptical or flexible cylindrical structures. The fibres of the gel are represented as a flexible worm-like chain of cylindrical Kuhn segments, as discussed previously^47, 57^. Uncertainties were calculated by the fitting software and the data were optimized starting from values determined from our previous data^32^.

### Photoconductivity measurements

Films of the xerogels for conductivity measurements were prepared using the method described previously^19, 33^. A small amount of the gel was placed onto a glass microscope slide with a 3 mm × 3 mm mask and allowed to dry in air overnight. When the sample was dry, the mask could be removed and two silver electrodes spaced 3 mm apart were added ensuring they had contact with the xerogel. The silver electrodes were made using silver paste (Electrodaag, Agar Scientific) which attached copper wired to the glass slide. Epoxy resin glue was placed over the silver electrodes to prevent oxidation of the silver in air. Again, this was left to dry overnight before measurements.

The conductivity measurements were also carried out as described previously^19, 33^ using an Autolab Potentiostat operating in a two-electrode configuration in the absence of a supporting electrolyte. A 365 nm, 400 nm, 450 nm, 470 nm, 528 nm, 590 nm and 628 nm LEDs (LedEngin Inc, LZ1-10U600) with a light source powered by a TTi QL564P power supply operating at 3.9 V were used as a light supply. Dark experiments were performed in a Faraday cage in air. Linear sweep measurements were recorded from −4 V to 4 V at a scan rate of 0.05 V/s and a preconditioning step at 0.002 V for 2 seconds. The current recorded at 4 V was then used as a value for photoresponse value at each wavelength. After irradiation of a film the photocurrent was allowed to decay completely, as confirmed by no current detected in the dark, before irradiating the film at a different wavelength.

The data generated or analysed during this study are included in this published article (and its Supplementary Information files) or are available from the corresponding author on reasonable request.

## Electronic supplementary material

Supplementary Information
