# Supplementary material for: Self-sorted Oligophenylvinylene and Perylene Bisimide Hydrogels
Source: Sci Rep. 2017 Aug 21;7:8380. doi: 10.1038/s41598-017-08644-0 (PMC5566499; doi:10.1038/s41598-017-08644-0)
Supplement: Supplementary file 1 — Supplementary Information [file 41598_2017_8644_MOESM1_ESM.pdf]

# Self-sorted Oligophenylvinylene and Perylene Bisimide Hydrogels

Ana M. Castilla,[a] Emily R. Draper,[a,b] Michael C. Nolan,[a,b] Christopher Brasnett,[c] Annela Seddon,[c,d] Laura L. E. Mears,[a] Nathan Cowieson[e] and Dave J. Adams[a,b]*

[a] Department of Chemistry, University of Liverpool, Crown Street, Liverpool, L69 7ZD, U.K.

[b] School of Chemistry, WestCHEM, University of Glasgow, Glasgow, G12 8QQ, U.K.

[c] School of Physics, HH Wills Physics Laboratory, Tyndall Avenue, University of Bristol, BS8 1TL, U.K.

[d] Bristol Centre for Functional Nanomaterials, HH Wills Physics Laboratory, Tyndall Avenue, University of Bristol, BS8 1TL, U.K.

[e] Diamond Light Source Ltd, Harwell Science and Innovation Campus, Didcot, OX11 0QX, U.K.

# Supporting Information

## Supporting Figures

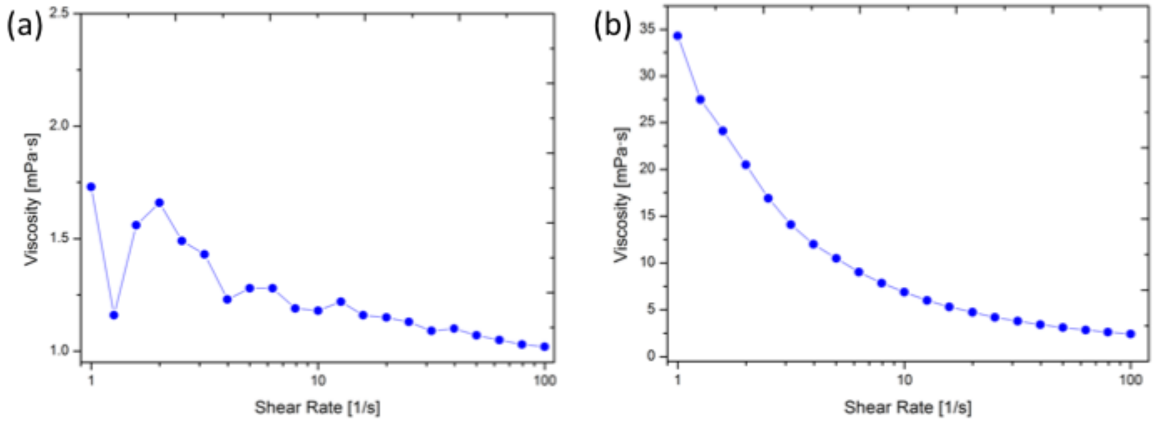


**Figure S1.** Viscosity measurement of an aqueous solution of (a) **1** + **3** and (b) **2** + **3** under increasing shear rate. Total concentration of gelator was 10 mg/mL, pH = 11.

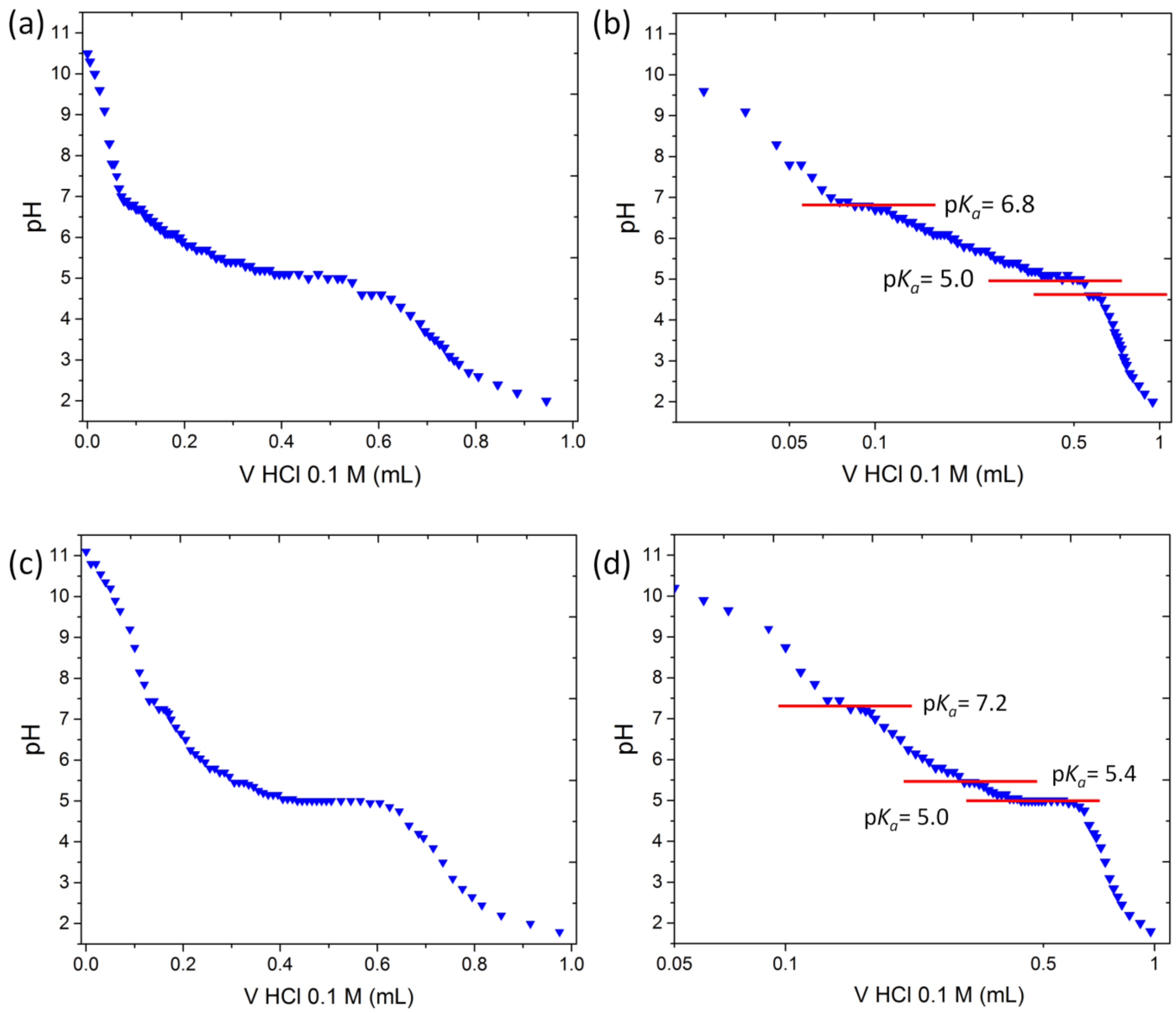


**Figure S2.** Top: Titration of a solution of **1+3** (1:1) with HCl **(**0.1 M**)** plotted on (a) a linear and (b) a logarithmic scale. Bottom: Titration of a solution of **2+3** (1:1) with HCl **(**0.1 M**)** plotted on (c) a linear and (d) a logarithmic scale.

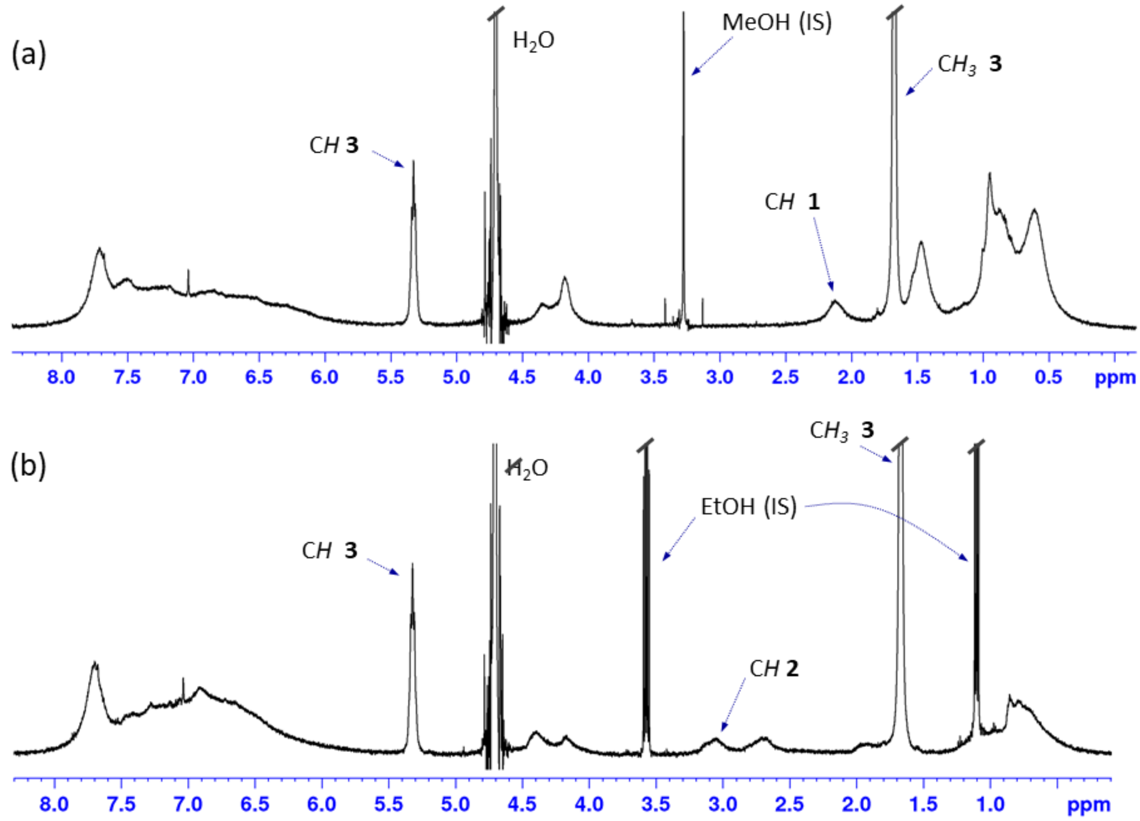


**Figure S3.** (a) $^1$H NMR of a 1:1 mixture of **1**+**3** in $D_2O$ (pH = 10.3) before the addition of GdL. The concentration is 5mg/mL of each gelator. MeOH was added as internal standard (IS); (b) $^1$H NMR of a 1:1 mixture of **2**+**3** in $D_2O$ (pH = 11). The concentration is 5mg/mL of each gelator. EtOH was added as internal standard (IS). The broadness of the peaks is indicative of the degree of aggregation. All the gelators here are assembled to some degree at pH 11, but 1 and 2 are significantly more aggregated than 3 at this pH. Hence, their peaks are broader.

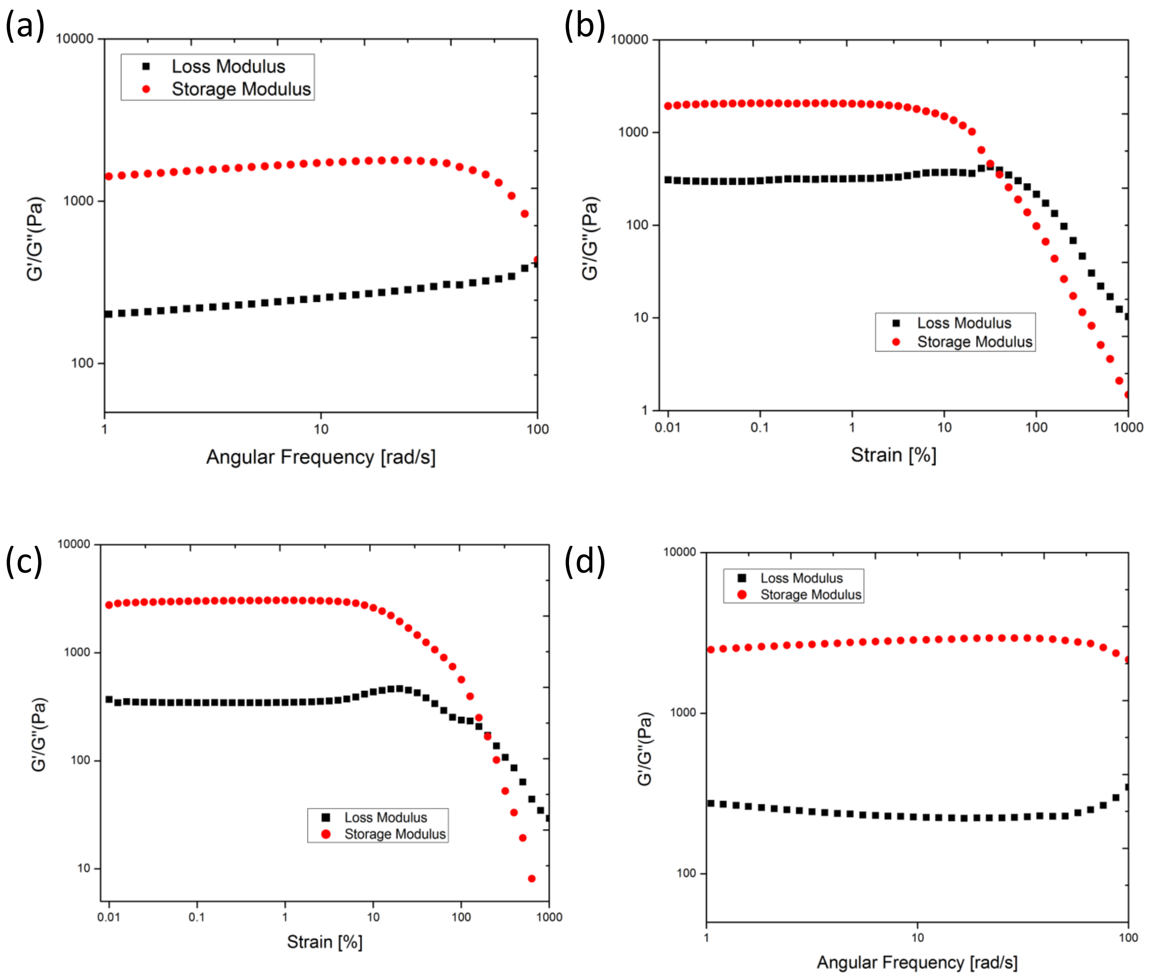


**Figure S4.** (a) Strain sweep for a mixed gel of **1**+**3**; (b) Frequency sweep for a mixed gel of **1**+**3**; (c) Strain sweep for a mixed gel of **2**+**3**; (d) Frequency sweep for a mixed gel of **2**+**3**. Full red circles represent G′ and full black squares represent G″.

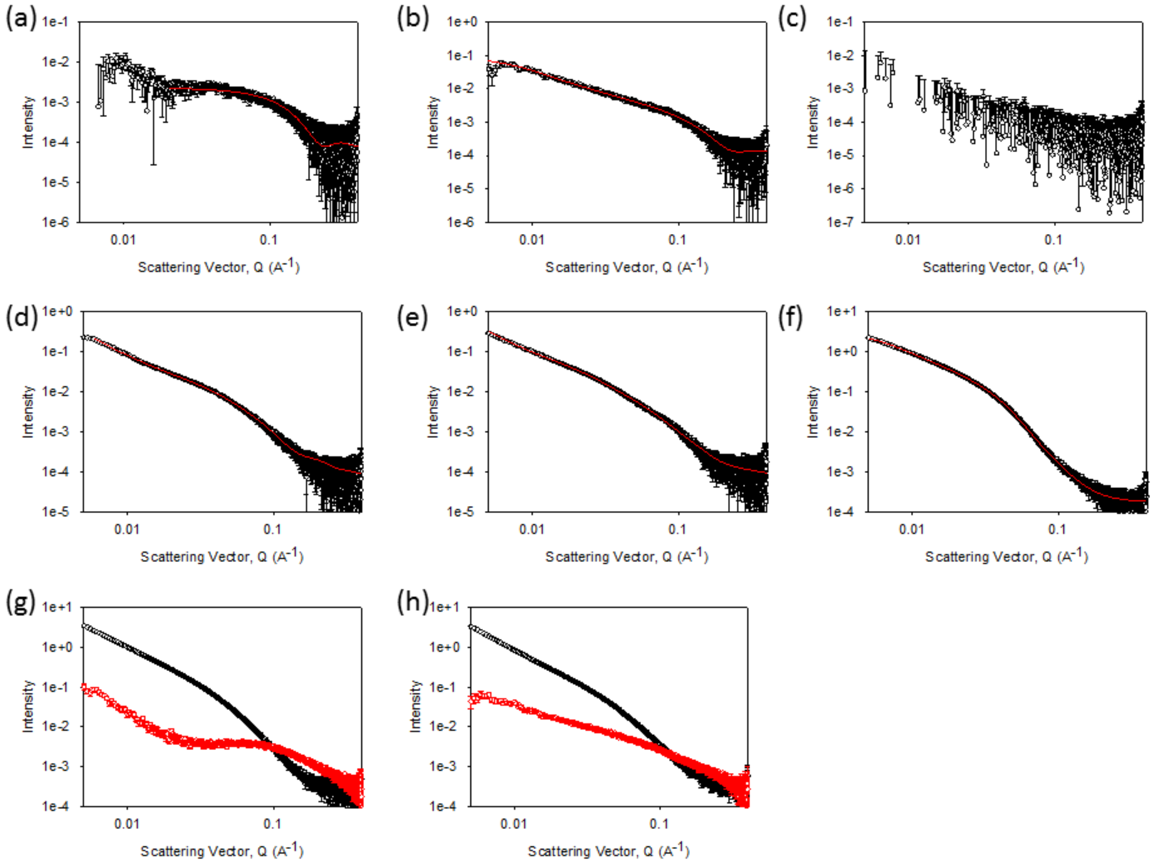


**Figure S5.** SAXS data and fitting. The scattering is shown for (a) a solution of **1**; (b) a solution of **2** at 5 mg/mL; (c) a solution of **3** at 5 mg/mL; (d) a gel of **1** at 5 mg/mL; (e) a gel of **2** at 5 mg/mL; (f) a gel of **3** at 5 mg/mL. In all cases, the data are shown in black and the fits (as discussed in the main text) are shown in red. For (a), the fit was cut off at 0.03 $Å^{-1}$ based on the shape of the data, to show the fit to the sphere component. Fitting to data at lower Q values lead to significantly worse fits (as shown by the chi value) to the spherical data. (g) overlays of the scattering from a solution of **1** + **3** (5 mg/mL of each component) immediately after adding GdL (red) and after 800 minutes (black). (h) overlays of the scattering from a solution of **2** + **3** (5 mg/mL of each component) immediately after adding GdL (red) and after 800 minutes (black). The fits to the data were performed based on initial values suggested by our previous small angle neutron scattering data.[1]

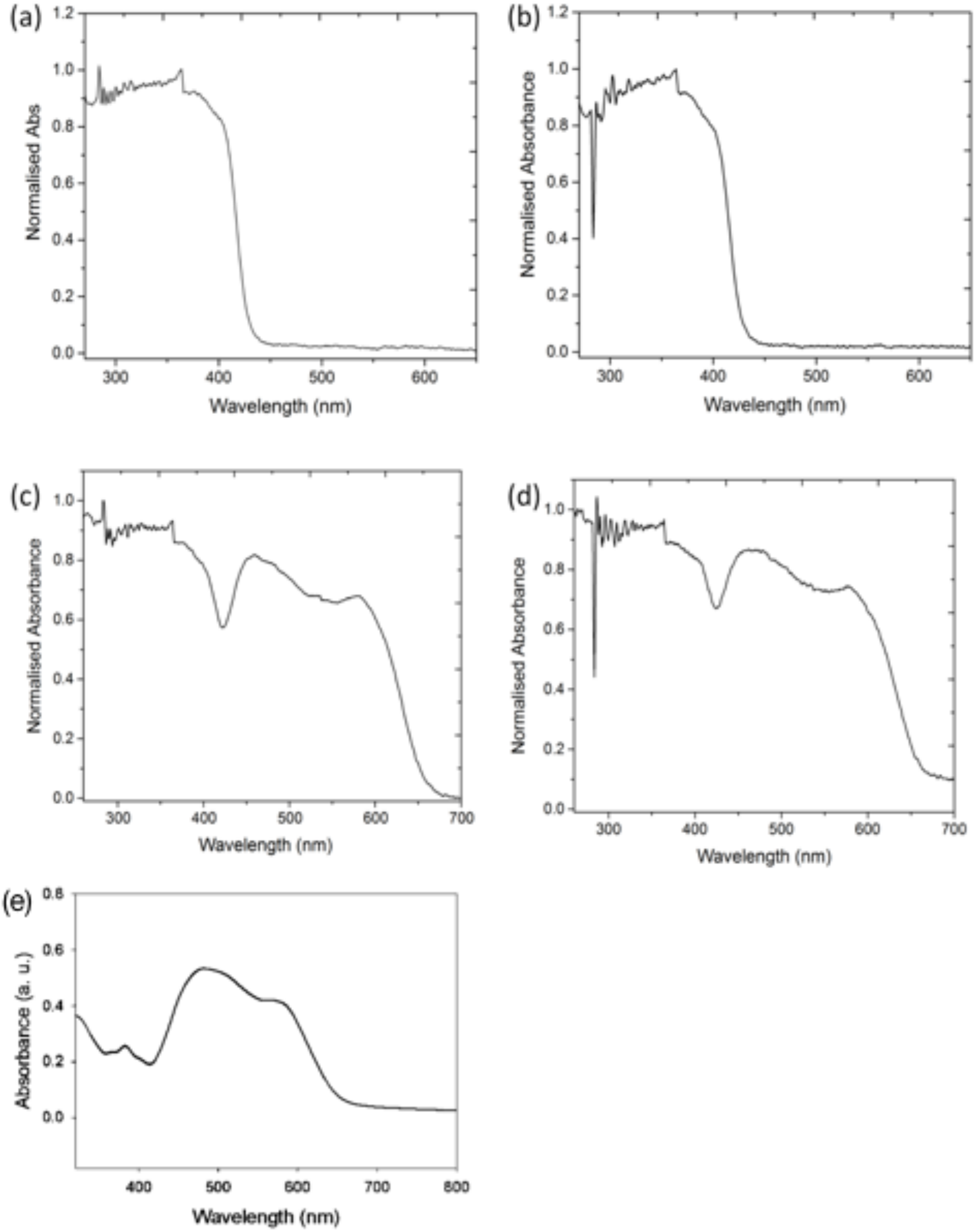


**Figure S6.** UV-Vis spectra of (a) a gel of **1**; b) a gel of **2;** (c) a gel of **1**+**3**; (d) a gel of **2**+**3**.; (e) a gel of **3** (previously shown in ref 2).

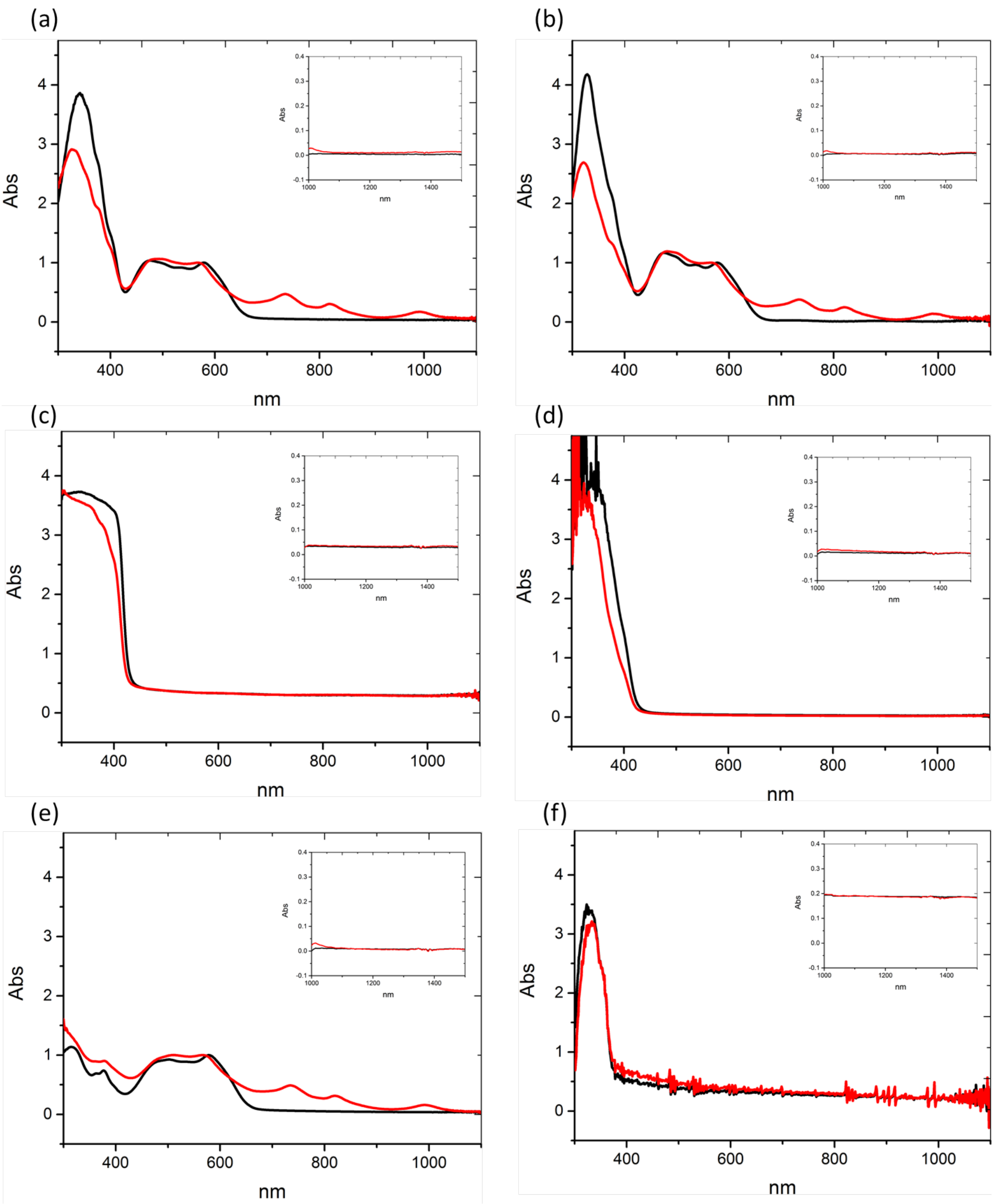


**Figure S7.** UV-Vis absorption spectra of xerogels before (black) and after (red) 365 nm irradiation. (a) **1**+**3**; (b) **2**+**3**; (c) **1**; (d) **2**; (e) **3**; (f) St-F (see Fig. S10 for structure). Inset spectra show the scans from wavelengths 1500-1000 nm. Samples were irradiated for 10 minutes.

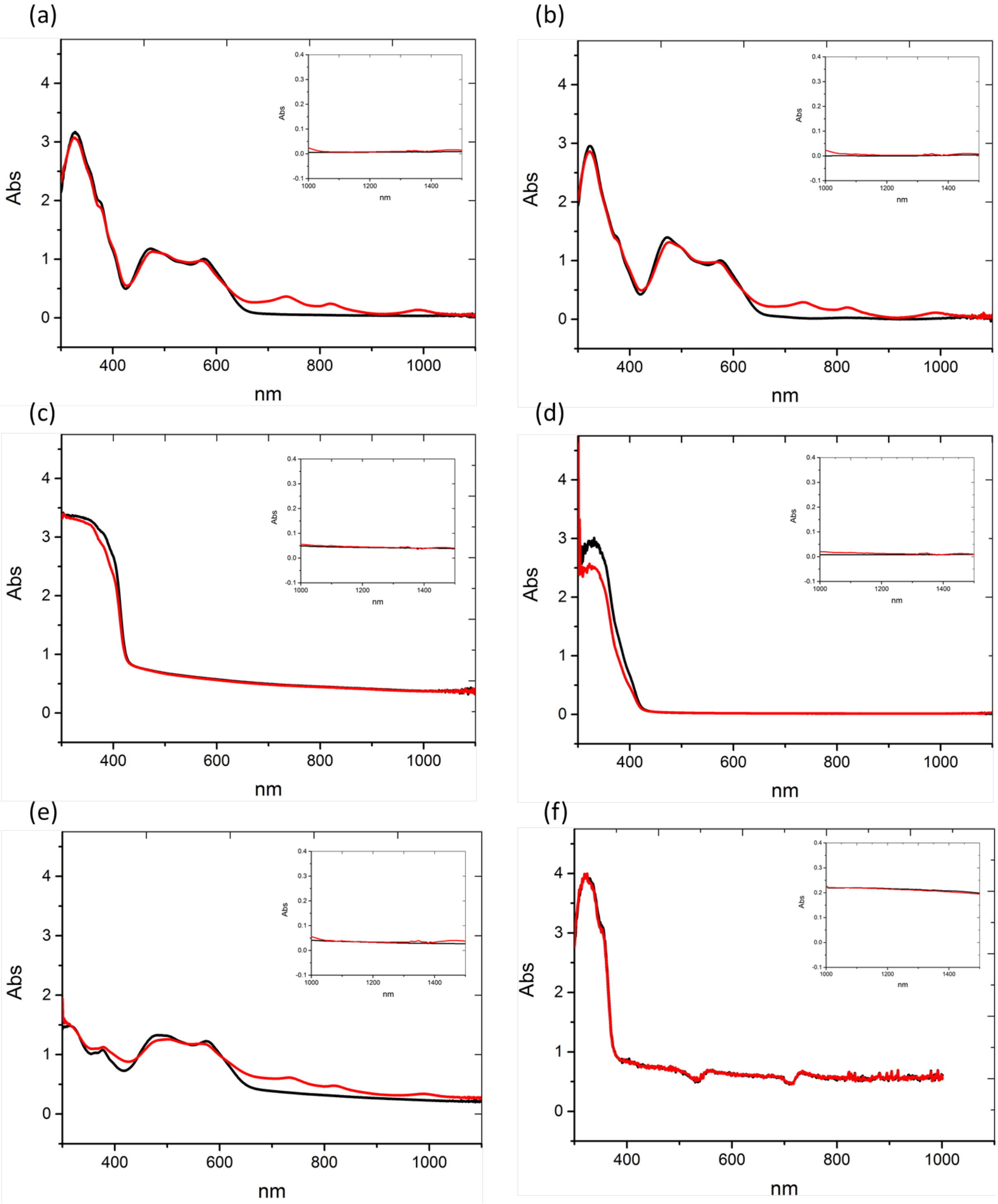


**Figure S8.** UV-Vis absorption spectra of xerogels before (black) and after (red) 400 nm irradiation. (a) **1**+**3**; (b) **2**+**3**; (c) **1**; (d) **2**; (e) **3**; (f) St-F (see Fig. S10 for structure). Inset spectra show the scans from wavelengths 1500-1000 nm. Samples were irradiated for 10 minutes.

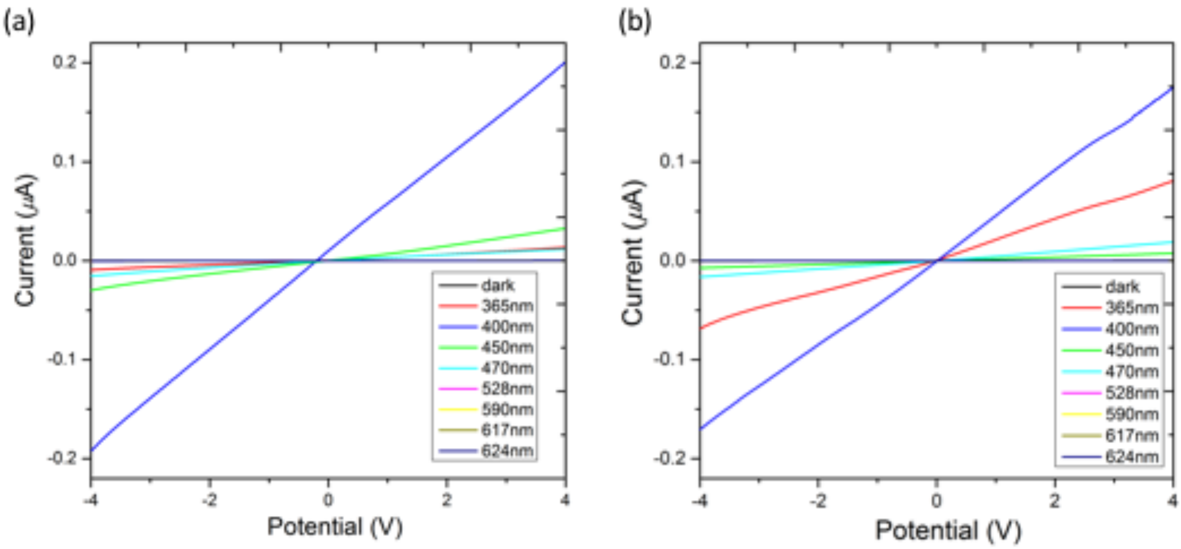


**Figure S9.** (a) Plot of I-V curve for a xerogel of **1**+**3** under different irradiation wavelengths and in the dark; (b) Plot of I-V curve for a xerogel of **2**+**3** under different irradiation wavelengths and in the dark.

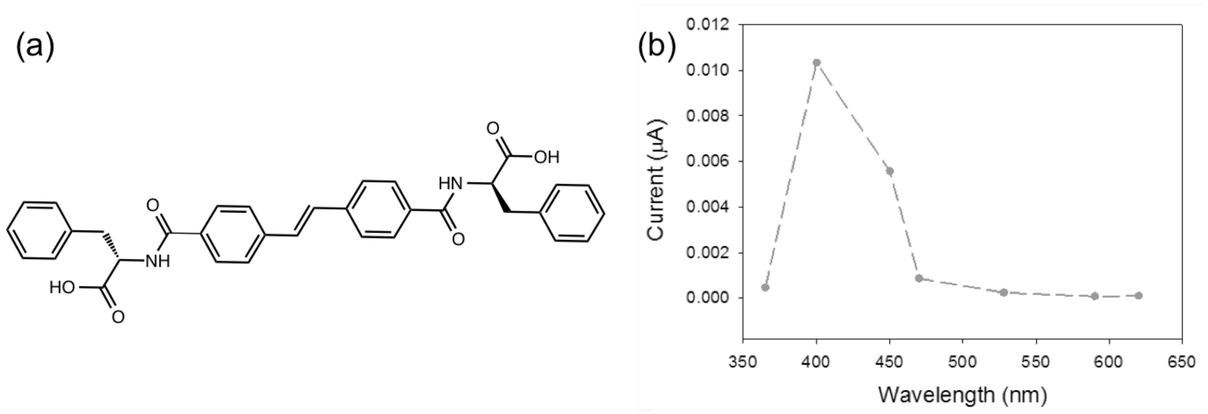


**Figure S10.** Chemical structure of the stilbene based gelator (b) Wavelength response in terms of absolute photocurrent measured at 4 V of a 1:1 mixture of the stilbene-based gelator with **3**.

## References


1. A. M. Castilla, M. Wallace, L. L. E. Mears, E. R. Draper, J. Doutch, S. Rogers and D. J. Adams, *Soft Matter*, **2016**, *12*, 7848-7854.
2. E. R. Draper, J. J. Walsh, T. O. McDonald, M. A. Zwijnenburg, P. J. Cameron, A. J. Cowan and D. J. Adams, *J. Mater. Chem. C*, **2014**, *2*, 5570-5575
